# *Ifugao *males, learning and teaching for the improvement of maternal and child health status in the Philippines: an evaluation of a program

**DOI:** 10.1186/1471-2458-11-280

**Published:** 2011-05-07

**Authors:** Noriko Kadomoto, Hajime Iwasa, Miyako Takahashi, Marcelyn M Dulnuan, Ichiro Kai

**Affiliations:** 1 The University of Tokyo, School of Public Health, Tokyo, Japan; 2 Dokkyo Medical University, School of Medicine, Department of Public Health, Tochigi, Japan; 3 Japan International Cooperation Agency, Ifugao, Philippines

## Abstract

**Background:**

Improving Maternal and Child Health (MCH) is a prioritized global agenda in achieving the United Nations Millennium Development Goal 5. In this challenge, involving males has been an important agenda, and a program with such intent was conducted in *Alfonso Lista, Ifugao*, of the Philippines. The objectives of this study were: (1) to evaluate the effectiveness in knowledge, attitude, and practice before and after a MCH session; (2) to evaluate the session's effectiveness in relation to socio-demographic characteristics; and (3) to examine if males who have learned about MCH topics can teach another group of males.

**Methods:**

A male community representative who received a lecture from the health office staff was assigned to teach a group of community males [Group 1, N = 140] in 5 sessions, using educational materials. 10 male volunteers from Group 1 then taught a different group of males [Group 2, N = 105] in their own *barangays *(villages). To evaluate its effectiveness, a self-administered questionnaire survey pertaining knowledge, attitude and practice regarding MCH was conducted at three different time points: before the session (Time 1, T1), after the session (Time 2, T2), and 3 months following the session (Time 3, T3). A repeated measures analysis of variance was conducted to test for changes over time and its interaction effect between specific socio-demographic variables.

**Results:**

In Group 1, there was a significant positive increase in knowledge score over time at T1-T2 and T1-T3 (*p *< 0.001). For attitude, the score increased only at T1-T2 (*p *= 0.027). The effectiveness in knowledge and attitude did not vary by socio-demographic characteristics. As for practice, majority of the participants reported that they had talked about MCH topics in their community and assisted a pregnant woman in some ways. A comparison between Group 1 and Group 2 revealed that Group 2 had similar effectiveness as Group 1 in knowledge improvement immediately after the session (*p *< 0.001), but no such improvement in the attitude score.

**Conclusion:**

Although the change in attitude needs further assessment, this strategy of continuous learning and teaching of MCH topics within community males is shown to improve knowledge and has a potential to uplift the MCH status, including the reduction of maternal deaths, in *Alfonso Lista, Ifugao, Philippines*.

## Background

Improvement of Maternal and Child Health (MCH) is a global agenda in achieving the United Nations Millennium Development Goal 5, which aims for a 75 per cent reduction of maternal deaths between 1990 and 2015. However, progress in reducing Maternal Mortality Ratio (MMR, deaths per 100,000 live-births) worldwide has been modest, with a decline of only 5.4% as of 2005. More than 530,000 mothers, 99% of them in developing countries, die each year and Asian countries account for nearly half of these deaths [1]. The leading cause of mortality is hemorrhage followed by anemia and sepsis/infection [2], which can be prevented and reduced by appropriate care. Some of the factors behind this global issue are reported to be poverty, socio-cultural factors, quality of care, and access to health services [3,4]. The MMR of the Philippines has gradually declined from 209 in 1990 to 162 in 2006 [5]. The FOURmula ONE for Health (2005-2010) covers four pillars including finance, regulation, service delivery, and governance to improve service efficiency, effectiveness, and equity. In this framework, the Safe Motherhood Policy promotes facility-based deliveries with a skilled birth attendant. However, the Philippines is still lagging behind in achieving the MDG 5, which requires a reduction of MMR to 52 by the year 2015.

Many health promoters see their role as protecting women from the impact of men's behavior and have worked directly with women to empower them [6]. Therefore, MCH issues have been seen as women's concerns, and health education programs have been focused on and directed toward women for many years [7-11]. However, male responsibilities and participation to promote gender equality was called upon at the International Conference on Population and Development held in Cairo (1994). Action Plan 4.27 stated that "special efforts should be made to emphasize men's shared responsibility and promote their active involvement...in maternal and child health" and Action Plan 8.22 recommended the "development of programs and education to engage men's support for maternal health and safe motherhood" [12]. These ideas were then refined at the Fourth World Conference on Women in Beijing (1995).

Yet, there have been few published evaluations of intervention that involves males in MCH [6]. Previous studies of male participation in women's reproductive health issues have focused in areas such as family planning [13-15] and sexually transmitted diseases including HIV/AIDS [16,17]. One of the few studies regarding MCH topics shows that not more than half of the male respondents could name one danger sign during pregnancy and delivery in India [18]. Also, males who received information had greater knowledge of the importance of antenatal care services and their partners made more visits to antenatal care clinics [6]. Furthermore, pregnant women who received education with their husbands were more likely to make three or more birth preparations and attend post-partum visits in Nepal [19]. There are also some studies on breastfeeding, where a male partner's knowledge is associated with positive changes in attitude and support for women's decisions to breastfeed [20-22].

A large number of the Knowledge Attitude Practice (KAP) studies relating to male participation in MCH are cross-sectional studies and do not look at its change over time. However, considering the fact that health education should be a continuous process in providing essential health knowledge, assuring the formation of sound attitudes relative to it, and securing desirable health behavior [23], an evaluation from such a perspective is necessary. Therefore, the first objective of this study was to evaluate the effectiveness in knowledge, attitude, and practice by comparing the outcome measures before and after an MCH session.

A study on health education materials, discusses the importance of considering the difference in age and educational background of its target, as information must be presented according to individual's knowledge, interests and ways of organizing knowledge [24]. Therefore, an intervention effect could vary by socio-demographic characteristics, and effectiveness must also be assessed and analyzed by these factors as well. Thus, the second objective was to evaluate the session's effectiveness in improving knowledge and attitude in relation to socio-demographic characteristics.

Health education can influence the community at the grass-roots level, either through formal or informal means. Studies recommend the involvement of religious leaders [25], peer-leaders [26], or supervisors [27] for greater impact through their political commitment and leadership to the target group. However, no studies have examined how health information may be handed over from one influential group to another by evaluating both sides, which is the key to accurate health information dissemination. This leads to the final objective of this study, which was to examine if community representatives who have learned about MCH topics can teach another group of males.

## Methods

### Program Overview

In 1995, the Empowerment and Reaffirmation of PATernal abilities (ERPAT) program was nationally developed and implemented by the Department of Social Welfare and Development. The purpose was to train male representatives to be an advocate in teaching other community males about family violence, drug-free home, family spirituality, etc. Early in the year 2009, the *Ifugao *Provincial Government in partnership with the Japan International Cooperation Agency (JICA) MCH Project and other development partners, localized this as a new strategy called the "Active Males Movement against violence and for AYOD (AMMA)." The AYOD Community Health Team, formerly called the Women's Health Team, is a voluntary community organization that conducts health promotion activities and had expanded its membership to males in 2007. *Ayod *is an *Ifugao *term for hammock, which is sometimes used for carrying sick people and pregnant women to the health facility over difficult terrain. This localized name was given to unify all the *Ifugaos*, including the males, to help one another as a hammock cannot be carried alone. This AMMA strategy, which had incorporated MCH topics to the former ERPAT programs, is referred to as a "Movement" because it involves males learning and teaching what they had learned to other males. The goals of this innovative strategy are to: (1) empower males by developing a positive concept of self; (2) develop knowledge, attitude, and behaviors on reproductive health; and (3) establish support networks among males in the community by enhancing their active participation in promoting a healthy lifestyle. This research is a feasibility study in line with the goals of this AMMA strategy.

### Study Site

This study was conducted in *Ifugao *Province, which is located in north central Luzon of the Philippines. According to the Field Health Service Information System (*Ifugao *Report), the MMR declined successfully from 194 in 1990 to 67 in 2008. However, among the 619 live births in 2008, 53.5% were still home-based deliveries and one maternal death occurred in 2009 at *Alfonso Lista *(population of 25,342 with 20 *barangays*), one of the 11 municipalities of *Ifugao*. For over years, the JICA and other partners have been supporting the upgrading of facilities, human resource development, and monitoring systems.

### Recruitment and Study Design

**Figure 1:**
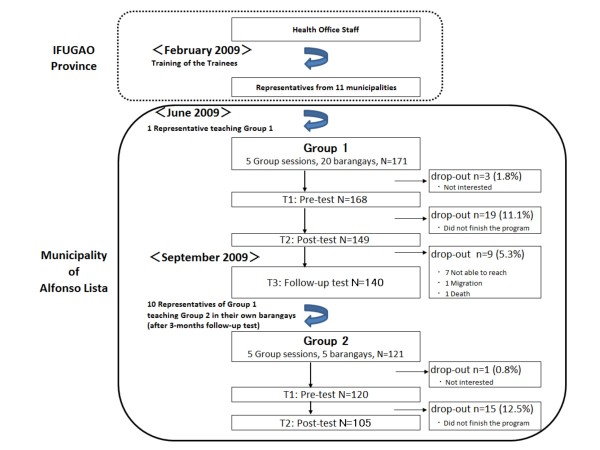
**Recruitment Flow and Study Design**.

The participants were recruited through the AYOD Community Health Team members and the inclusion criteria were males over the age of 18 (Figure 1).

In February 2009, one male community representative received training from the health office staff. Then in June, he was assigned to teach five groups of males on MCH topics, shown in Table 1, at the nearby *barangays *(Group 1). Each group session, which took approximately five hours, included a PowerPoint presentation and participatory learning activities in which participants shared their knowledge and experiences. The educational materials were written in lay English, but both English and the local dialect of *Ilocano *were used during the PowerPoint presentation, which was prepared by a public health nurse. The lecturer read the topics in English, but the explanation was mostly in *Ilocano*, for the better understanding of the participants. Technical assistance was given by a public health nurse, as required. In September, ten male volunteers from Group 1, in pairs of two, taught another different group of males in their own *barangays *(Group 2). They used the same educational materials and had the same timeframe as with the prior groups. Two health-related research assistants validated that all the basic content had been covered and discussed by the lecturers, using a check-list. During the study, socio-demographic characteristics, knowledge, attitude, and practice of these two groups of males were collected through a self-administered questionnaire survey. To evaluate the effectiveness, pre-test (Time 1, T1: before the session), post-test (Time 2, T2: immediately after the session), and follow-up test (Time 3, T3: three months following the session) were conducted. Surveys were given at all times for Group 1, whereas for Group 2, they were only given at T1 and T2.

**Table 1:** Topics on Maternal and Child Health

Pre-natal	Early signs of pregnancy	Common discomforts during pregnancy
	Importance of pre-natal care	Ways to ease discomforts
	Check-up schedules	Serious danger signs during pregnancy
	(number of times and when)	Things to bring for facility delivery
	Available services at a health facility	Birth Planning
	Tetanus toxoid immunization	Arrangements by the family for facility-delivery
**Delivery**	Advantages of health facility-based delivery	

**Post-partum**	Serious complications after birth	
	Importance of post-partum care	
	Good post-partum care practices	

**Three delays model**	First, second, and third delay	

In Group 1, out of 171 males who participated in the program, a total of 168 agreed to participate in this study by answering the pre-test. Then, a total of 149 participants also answered the post-test and 140 males were followed up 3 months after the session. The loss in tracking was mainly due to busy schedule in farm work, living in a far away hard-to-reach catchment, migration, and death after the intervention. In Group 2, out of 121 program participants, 120 males answered the pre-test and 105 of these males completed the post-test. The loss in number was due to family matters and farm work, thereby making them leave before completing the session and answering the questionnaire. No monetary incentives were given to the participants, including the transportation fee, but a lunch and a snack with beverages were provided.

### Outcome Measures

A structured questionnaire developed by the researcher, with questions on knowledge, attitude and behavior regarding MCH, was administered. Socio-demographic data such as age, occupation, education, and average income were also collected. The questionnaire was pilot tested, with ten *Ifugao *males, to determine whether the items were adequate, clear, and culturally appropriate. Minor revisions were applied by rephrasing some of the questions.

Knowledge was measured by 17 questions with 5 items each (Cronbach α = 0.87), asking how well the participants knew about MCH topics. The questions were based on the educational materials used during the session, with a range of 0-85: higher score indicating better knowledge. The questions included, 'What are the early signs of pregnancy?', 'What is Birth Plan?', and 'What are good post-partum care practices?' The answers were in multiple-choice, and the participants encircled the answer(s) of their choice. The total score was calculated for analysis by giving 1 point for each correct answer and no points for 'Don't know' answers.

Attitude was evaluated through 10 items (Cronbach α = 0.86), measuring how strongly they felt towards helping women as individuals, being a male in general, and being a community member. To measure the intensity of their attitudes, a scale from 'strongly disagree' to 'strongly agree' was used. The range was 1-5, with the most desirable attitude calculated as 5, and its mean was calculated. The items included, 'I should help women deliver at a health facility', 'In general, males have a big role in supporting women through pregnancy, delivery, and after-birth', and 'I can be a model/example to other *Ifugao *males in helping safe pregnancy, delivery, and after-birth.'

Practice was measured by asking for their experiences such as 'Discussing about MCH topics' and 'Assisting women in times of pregnancy', within the three months between T1 and T3. The answer choices were 'yes' or 'no' with further open-ended questions.

### Statistical Analysis

Data analysis was performed using the software package SPSS ver. 18.0 and SAS ver. 9.1. The baseline socio-demographic differences between the two groups were compared using the Student's *t*-test or chi-square test. A repeated measures analysis of variance was conducted to test for changes over time and its interaction effect between specific socio-demographic variables. The difference between the group sessions in Group 2, with different lecturers, was considered as a random effect in a mixed effect model. *P*-values of < 0.05 were considered to be statistically significant.

### Ethical Considerations

All procedures were reviewed and approved by the Institutional Review Board of the Graduate School of Medicine, The University of Tokyo (No. 2576). The participants were informed about the study objectives and procedures, and signed the "Informed Consent Form" prior to enrolment.

## Results

A total of 140 (Group 1) and 105 (Group 2) males fully participated in the study, representing 81.9% and 87.5% completion rate, respectively (Table 2). The drop-outs (n = 28) in Group 1 were younger (*p *= 0.021) and had lower knowledge scores (*p *< 0.001) compared to the participants. In Group 2, the knowledge scores of the drop-outs (n = 15) were lower (*p *< 0.001) than the participants.

**Table 2:** Participants' Characteristics

	Group1 (N = 140)		Group 2 (N = 105)		*p*
**Age**					
Range		18-71		18-74	
Mean (SD)		42.5 (± 11.3)		38.0 (± 12.2)	*
18-19	2	1.4%	5	4.8%	
20-29	18	12.9%	22	21.0%	
30-39	34	24.3%	38	36.2%	
40-49	51	36.4%	20	19.0%	
50-59	25	17.9%	14	13.3%	
60-69	8	5.7%	3	2.9%	
70-79	2	1.4%	2	1.9%	
**Education**					**
Below High School Graduate	69	49.3%	79	75.2%	
High School Graduate and Above	71	50.7%	26	24.8%	
**Marital Status**					*
Married	131	93.6%	88	83.8%	
Others	9	6.4%	17	16.2%	
**Occupation**					ns
None	5	3.6%	2	1.9%	
Farmer	113	80.7%	94	89.5%	
Government Worker	14	10.0%	6	5.7%	
Others	8	5.7%	3	2.9%	
**Number of Family Members**					
Range		1-15		1-12	
Mean (SD)		5.5 (± 2.4)		5.4 (± 2.6)	ns
< 5	79	56.4%	61	58.1%	
6 ≦	61	43.6%	44	41.9%	
**Number of Children**					
Range		0-13		0-10	
Mean (SD)		3.7 (± 2.5)		3.2 (± 2.7)	ns
None	12	8.6%	19	18.1%	
1 - 3	101	72.1%	42	40.0%	
≦ 4	27	19.3%	44	41.9%	
**Planning to Have Children**					ns
Yes	65	46.4%	53	50.5%	
No	72	51.4%	52	49.5%	
**Monthly Income, Peso **(5000 pesos = about 100 USD)					ns
< 5,000	93	66.4%	81	77.1%	
5,000≦	47	33.5%	24	22.8%	
**Head of the Household**					ns
Myself	119	85.0%	83	79.0%	
Other than Myself	21	15.0%	22	21.0%	
**Decision-maker for using money on health**					ns
Myself	102	72.9%	75	71.4%	
Other than Myself	36	25.7%	28	26.7%	
**Heard About Maternal Death in January 2009**					ns
Yes	51	36.4%	47	44.8%	
No	89	63.6%	58	55.2%	

*t*-test or Χ^2 ^test

*: *p *< 0.05; ^**^: *p *< 0.001; ns: not significant

### Group 1: Change in Knowledge, Attitude, and Practice

Table 3 shows the changes in knowledge and attitude score within Group 1. There was a significant increase in overall knowledge at T1-T2 and T1-T3 (F = 54.97, *p *< 0.001). After analyzing this result per questions, 14 out of 17 showed this effect. However, the scores on 'Why should a mother go for pre-natal care?' increased significantly, while the scores on 'What are the things mothers need to bring from home for delivery at a health facility?' and 'What is "Birth Plan"?' decreased significantly at T2-T3. For overall attitude, the score increased significantly only at T1-T2 (F = 3.63, *p *= 0.027), and decreased slightly at T3 with no significant difference between T1-T3. During the three months after the session, many of the participants practiced what they had learned. Out of the 140 participants, 85% talked about MCH topics to other people in the community. Also, 79% said that they assisted pregnant women, such as their wives, sisters, daughters, daughter-in-laws, neighbors, and friends. And, some of the ways that they have mentioned were: (a) referring to the midwife or doctor; (b) offering transportation/money; (c) advising to go to the hospital; (d) going to their house to take care of the children and livestock; and (e) including them in prayers.

**Table 3:** Group 1: Changes in Knowledge and Attitude Scores

	Time				
			
	T1	T2	T3	**F**	*Tukey*
	(Pre-test)	(Post-test)	(3-months follow up)		
			
	Mean (SD)				
**KNOWLEDGE**					
(0-85, the highest is 85)	36.3 (± 15.3)	51.7 (± 13.4)	51.2 (± 13.1)	54.97*	T1-T2, T1-T3
**ATTITUDE**					
(1-5, the highest is 5)	3.99 (± 0.6)	4.17(± 0.5)	4.05 (± 0.6)	3.63*	T1-T2

repeated measures analysis of variance

*p < 0.05

### Group 1: Change in Knowledge and Attitude by Socio-demographic Characteristics

The effectiveness of the intervention in knowledge and attitude was analyzed by specific socio-demographic variables listed in Table 2. There was no significant interaction effect in any of the variables, demonstrating that effectiveness did not vary regardless of these socio-demographic characteristics. The variables that had significant main effects were education (F = 38.99, *p *< 0.001), occupation (F = 8.64, *p *< 0.001), monthly income (F = 9.84, *p *= 0.002), and household-head (F = 7.43, *p *= 0.007) for knowledge, and education (F = 13.763, *p *< 0.001) for attitude (Table 4).

**Table 4:** Group 1: Changes in Knowledge and Attitude Score by Socio-demographic Variables

	Time						
					
	T1	T2	T3	Time	Time*Group	Group	Tukey
	(Pre-test)	(Post-test)	(3-months follow up)				
			
	Mean (SD)			F			
**KNOWLEDGE**							

**Occupation**							
None	40.0 (± 8.5)	49.2 (± 10.13)	52.0 (± 10.58)	13.63 *	0.33	8.64 *	T1-T2, T1-T3
Farmer	34.5 (± 15.4)	49.9 (± 12.97)	50.2 (± 13.53)				Farmer
Government Worker	43.8 (± 15.8)	60.1 (± 13.3)	55.5 (± 11.10)				- Government Worker,
Other	45.6 (± 9.24)	63.6 (± 11.88)	58.1 (± 9.88)				Farmer - Other
**Education**							T1-T2, T1-T3
< High School Graduate	31.6 (± 17.79)	46.5 (± 13.01)	48.6 (± 13.96)	60.12 *	1.31	38.99 *	< High School Graduate
High School Graduate≦	40.8 (± 10.85)	56.7 (± 11.93)	53.8 (± 11.83)				- High School Graduate≦
**Monthly Income, Peso **(5000 pesos = about 100 USD)							
< 5,000	35.4 (± 16.19)	49.8 (± 12.88)	49.4 (± 12.80)	53.34 *	0.51	9.84 *	T1-T2, T1-T3
5,000≦	37.9 (± 13.49)	55.4 (± 13.85)	54.8 (± 13.18)				< 5,000 - 5,000≦
**Household Head**							
	36.8 (± 15.99)	52.7 (± 13.68)	52.0 (± 13.11)	25.44 *	0.425	7.43 *	T1-T2, T1-T3
Other than myself	33.1 (± 10.61)	45.8 (± 10.30)	47.0 (± 12.79)				Myself - Other than myself

**ATTITUDE**							

**Education**							
< High School Graduate	3.87 (± 0.55)	4.05 (± 0.47)	3.93 (± 0.60)	3.79 *	0.00	20.78 *	T1-T2
High School Graduate≦	4.11 (± 0.63)	4.28 (± 0.42)	4.17 (± 0.51)				< High School Graduate - High School Graduate≦

repeated measures analysis of variance

*p < 0.05

### Comparison between Group 1 and Group 2

**Figure 2:**
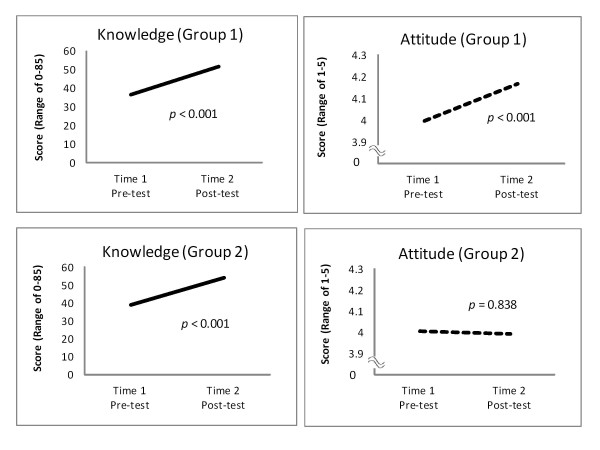
**Comparison of Change in Knowledge and Attitude between Group 1 and Group 2**. Paired t-test (Group 1); Mixed effect model, "session" as random effect (Group 2)

**Figure 3:**
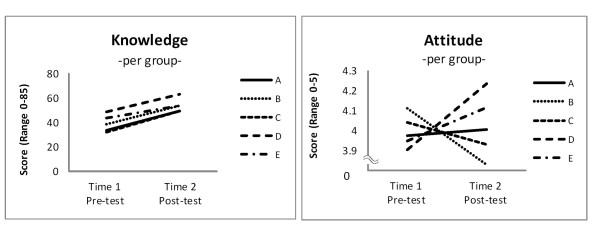
**Group 2: Change in Knowledge and Attitude by Sessions**. Adjusted for age, education, and marital status

A comparison of socio-demographic characteristics between Group 1 and Group 2, showed difference in age, education, and marital status (Table 2). Figure 2 shows differences in change in knowledge and attitude of the two groups at T1-T2. There was a significant increase in knowledge within both Groups 1 and 2 (*p *< 0.001). However, with attitude, there was a significant increase in Group 1 (*p *< 0.001), but no similar trend was observed in Group 2 (*p *= 0.838). A further analysis of Group 2 by different group sessions, which were held by different lecturers in various *barangays *(Figure 3), revealed that there was no significant interaction effect in knowledge (F = 0.846, *p *= 0.499), but attitude varied (F = 3.850, *p *= 0.006) even after having been adjusted for age, education, and marital status.

## Discussion

This study evaluated the effectiveness of teaching males about MCH topics in a community and if they can teach another group of males in the process of information dissemination.

The drop-outs in both of the groups with low baseline knowledge score, provide many implications for future program management. Giving them more clearer and direct messages as to why this program is important may have been necessary. For example, instead of an introduction with explanation of MDGs and the high maternal mortality rate of the Philippines, one may share stories of maternal death within the particular community; how and why mothers are losing their lives while giving birth. Also, findings in a systematic survey on women's views and experiences confirm that they prefer small-group learning environment in which they can talk to each other as well as the educator and can relate information to their individual circumstances [28]. In addition to having the lecturers lead the session without making the participants feel the content to be too difficult, this may be one of the ways to orient the program for lower drop-out rates.

The overall knowledge increased immediately after the session and was maintained even after three months. However, the scores on questions asking 'What are the things mothers need to bring from home for delivery at a health facility?' and 'What is Birth Plan?' decreased significantly at T2-T3, indicating that these topics might have required more emphasis. An effort in endorsing the importance of preparing for facility-based deliveries as males, and clarifying the difference between "Family Planning" and "Birth Planning", is perhaps necessary.

There was a significant increase in overall attitude score at T1-T2, followed by a slight decrease at T3 with no significant difference between T1-T3. Since the difference between T1-T3 was not significant, it is most likely that the session was not effective in changing participants' attitude over time. In the long *Ifugao *culture, females have been seen as the most responsible for child bearing. This may result in an unintentional negative perception on males' mind in terms of helping pregnant women, after three months following the session. This difficulty in changing male's attitude can be inferred by the description of an attitude as a belief that is shaped by forces that act upon it because of the position or status within social structure [29]. Various ways to motivate a community to develop a positive attitude must be further assessed, since it is an important aspect of health education in preserving and promoting health [30]. However, there remains a possibility that attitude change requires a longer duration, making it inconclusive in this short time.

In spite of the short three-month period after the session, many participants have acted as advocates in the community. They tried to share their learning with the people in their community and some even discussed it at the *barangay *assembly to raise community awareness regarding MCH issues. According to the study capturing fathers' conceptions of parental education topics, it is expected that it extend beyond the mother's role as the traditional infant caregiver to include the father's role as the other primary partner in providing infant care [31]. Therefore, this program of addressing MCH issues to males provides a good prospect for fathers wanting to help mothers improve their health status.

Findings from this study revealed that this strategy had similar effectiveness for any socio-demographic group of males. However, as for knowledge, main effects showed that public workers had higher scores than farmers, and males with higher educational background and higher monthly income tended to have better scores. These results coincide with findings from other studies, in which males with higher socio-economic status tend to know more about reproductive health matters [32]. However, the fact that it was equally effective regardless of their initial score at T1, is a satisfactory result for a first trial. In addition, being the head of a household was another factor that contributed to having a higher knowledge score. This might be explained by the responsibility that they have always possessed and the leading role that they have played in their family. For attitude, those with higher educational background had better attitude than those less educated. Taking in consideration of the fact that those with higher educational background also had higher scores in knowledge, there might be a relationship between knowledge and attitude, although the direction is unknown.

In a comparison between Groups 1 and 2, there was an increase in overall knowledge score at T1-T2, demonstrating that knowledge learned by one group of males can be taught to another group of males through this strategy. However, attitude score in Group 2 seemed to show a different trend compared to Group 1, with no significant positive change. Although it is difficult to conclude what factor contributed the most to this difference, one factor may be the teaching method since there were several lecturers that had been assigned for Group 2. Some of these lecturers had stated, "I felt nervous, did not expect myself to be so" and "I felt so nervous because this was my first time as a speaker." There is a need for training to enhance their teaching and facilitating skills, so they would feel more confident talking in front of a group and be successful in conveying the messages to the participants. Other factors such as the interaction between the participants and the lecturers and the tone of the lecturers could have also affected attitude change.

This study has several limitations. First, the recruitment of the participants was not through random sampling and leaves a possibility that they do not represent the community, making the findings difficult to generalize. Second, outcome measurements should be further assessed since they are not standardized nor validated. Since the pre-existing attitude scores were already generally positive, it may have reflected a selection bias that resulted in a ceiling effect. A more sensitive and reliable measurement for attitude should be developed for future studies. For practice, the measure did not take into consideration the difference in individuals in terms of actually having the opportunity to help women. A more concrete analysis can be done by following up the actual number of pre-natal and post-partum checkups and health facility-based deliveries that were supported by males. In the long term, MMR is also an important outcome measure for a better and complete understanding of the impact of this strategy. Lastly, for a more detailed analysis on the effect of this strategy, a comparison with sessions taught by health professionals, with female participation, or setting a control group must be considered. Also, factors such as the male participants' wives or household members should also be taken into account in future studies, as they could influence the male's knowledge, attitude, and practice.

## Conclusion

The WHO Making Pregnancy Safer initiative implies that availability of quality services alone will not produce the desired health outcomes when there is no possibility to be healthy, to make healthy decisions, and to be able to act on those decisions [33]. These community males that have acquired MCH knowledge and positive attitude towards helping women, can thus contribute to their community in many dimensions for the good of mothers' and children's health. Furthermore, it is noted that women, families, and communities must work more closely together with health care providers in achieving the reduction of mortality [33]. Along with each individual's participation, a strong political commitment and leadership, especially of the *barangay *officials is essential in making this bond between the health professionals and the community stronger. However, with this continuous learning and teaching strategy among the community males, there is a high potential for more improvement in the MCH status of *Alfonso Lista, Ifugao*, Philippines.

## Competing interests

The authors declare that they have no competing interests.

## Authors' contributions

NK conceived the study, collected the data, performed the data analysis, drafted and revised the manuscript. IK and MT contributed in the discussions for conceptualizing the study design and analysis methods. IK and HI participated in the interpretation of the results and discussions for manuscript writing and finalization. MMD supported in data collection and in coordinating for the study. All authors read and approved the final manuscript.

## Pre-publication history

The pre-publication history for this paper can be accessed here:

http://www.biomedcentral.com/1471-2458/11/280/prepub
